# The Efficacy of a Multimodal Bedroom-Based ‘Smart’ Alarm System on Mitigating the Effects of Sleep Inertia

**DOI:** 10.3390/clockssleep6010013

**Published:** 2024-03-18

**Authors:** Carolina Campanella, Kunjoon Byun, Araliya Senerat, Linhao Li, Rongpeng Zhang, Sara Aristizabal, Paige Porter, Brent Bauer

**Affiliations:** 1 Delos Living LLC, New York, NY 10014, USAlinhao.li@delos.com (L.L.); sara.aristizabal@delos.com (S.A.); 2 Well Living Lab, Inc., Rochester, MN 55902, USA; asenerat@gmail.com (A.S.); zhangrongpeng@hnu.edu.cn (R.Z.); paigemp@umich.edu (P.P.); 3 International Society for Urban Health, New York, NY 10003, USA; 4 School of Environment and Sustainability, University of Michigan, Ann Arbor, MI 48109, USA; 5 Department of Medicine, Mayo Clinic, Rochester, MN 55905, USA; bauer.brent@mayo.edu

**Keywords:** sleep inertia, vigilance, dawn simulation, temperature, sound, smart alarm, bedroom

## Abstract

Previous work has demonstrated the modest impact of environmental interventions that manipulate lighting, sound, or temperature on sleep inertia symptoms. The current study sought to expand on previous work and measure the impact of a multimodal intervention that collectively manipulated light, sound, and ambient temperature on sleep inertia. Participants slept in the lab for four nights and were awoken each morning by either a traditional alarm clock or the multimodal intervention. Feelings of sleep inertia were measured each morning through Psychomotor Vigilance Test (PVT) assessments and ratings of sleepiness and mood at five time-points. While there was little overall impact of the intervention, the participant’s chronotype and the length of the lighting exposure on intervention mornings both influenced sleep inertia symptoms. Moderate evening types who received a shorter lighting exposure (≤15 min) demonstrated more lapses relative to the control condition, whereas intermediate types exhibited a better response speed and fewer lapses. Conversely, moderate evening types who experienced a longer light exposure (>15 min) during the intervention exhibited fewer false alarms over time. The results suggest that the length of the environmental intervention may play a role in mitigating feelings of sleep inertia, particularly for groups who might exhibit stronger feelings of sleep inertia, including evening types.

## 1. Introduction

When individuals first wake up, they undergo a transitional period between sleep and wakefulness marked by drowsiness, disorientation, sleepiness, and temporarily impaired performance [1,2]. This impairment, called ‘sleep inertia’, has been observed across various cognitive domains, including working memory [3], vigilance [4,5], logic reasoning [6], and decision making [6,7]. Sleep inertia can occur after either a night of sleep, interrupted sleep at night, or a daytime nap and can last anywhere from one minute to up to three hours [2].

The severity of sleep inertia can depend on a few different biological factors, including the sleep stage upon awakening [4,8,9,10], variations in sleep architecture [4], prior sleep loss [10,11,12], and the circadian phase [5,13]. Seasonal changes in light during the winter can also increase the severity of sleep inertia due to a lack of morning light, which can either impact natural alerting mechanisms [14,15,16,17] or remove a phase-advancing light stimulus [18,19,20,21] that can potentially counter the effects of sleep inertia.

While sleep inertia is a fairly common phenomenon, it can have devastating consequences for those in professions with irregular sleeping schedules who are then required to make split-second decisions, such as individuals in healthcare and the transportation industry [22,23,24,25]. As a result, much research has been dedicated to investigating potential countermeasures, including consuming caffeine before naps [26,27] or immediately upon waking [28] and face washing [29]. In addition, a number of environmental interventions at different time-points (i.e., waking during the day and at night) have been explored, including manipulating lighting [29,30,31,32,33] and sound [34,35,36] and measuring the relationship with body temperature [37,38].

Attempting to counteract the effects of sleep inertia is particularly intriguing, given the relationship between the body and the environment. Previous research has shown that bright light can influence physiological changes in the body, including suppressing melatonin [39,40], increasing body temperature [41], and increasing cortisol levels [41]—all of which impact sleep and feelings of alertness. Likewise, sleep inertia is reported to be worse when individuals are woken up around the trough of the core body temperature cycle [4,42], though some contrary evidence suggests that the time of day does not matter [6]. Sleep inertia is also hypothesized to occur in part due to a decline in cerebral metabolism, which occurs from thermal downregulation during sleep [43], suggesting some relationship with body temperature.

To date, the outcomes of studies measuring environmental countermeasures are mixed due to methodological limitations such as the lack of a control group, no evidence of sleep inertia in the control group, or not enough testing points [44]. Additionally, factors such as light may be more effective when used at certain points, such as during nocturnal awakening [45,46]. However, despite some inconsistencies in outcomes, there have been some promising findings, which are summarized below.

With respect to lighting-based solutions for sleep inertia, Giménez et al. [31] found that an alarm clock with dawn simulation significantly reduced complaints of sleep inertia, though the mechanism for how this occurred was unclear. Exposure to polychromatic short-wavelength-enriched light immediately after waking from slow-wave sleep at night was shown to potentially improve subjective alertness, mood, and vigilant attention [32]. In another study, saturated red light delivered to individuals while their eyes were closed was shown to help mitigate sleep inertia upon waking [30]. Similar benefits have been observed in studies manipulating temperature, where one study demonstrated that applying a cold wet cloth or a fan breeze improved post-nap performance [47]. With respect to sound, a promising study found that exposing participants to pink noise after a 1-h nap in the afternoon eliminated the sleep inertia effects observed in the no-noise control group [36].

However, despite some encouraging findings, the positive effects observed in single environmental interventions on sleep inertia symptoms are modest. One solution could be to combine different environmentally based interventions. Single environmental interventions on their own might not be sufficiently powerful to impact sleep inertia, whereas the combined effect of manipulating lighting, temperature, and sound may provide ample influence on physiological processes to result in a lasting alerting influence. To date, no study has looked at the efficacy of a comprehensive environmental intervention where various aspects of the bedroom environment are manipulated to reduce sleep inertia complaints. The passive nature of such an intervention might also increase its adoption as, after the initial setup, little effort is needed from the individual. Therefore, the goal of the current study was to measure the impact of a multimodal alarm system in the bedroom that manipulated lighting, ambient temperature, and sound to mitigate the symptoms of sleep inertia as measured by performance on a vigilance task and subjective report.

## 2. Results

### 2.1. Impact of Intervention on Sleep Inertia Symptoms

To measure the impact of the bedroom-based alarm intervention, we tested vigilance (as measured by the performance on various PVT metrics (the mean reaction time, mean response time, percentage of 355 ms lapses and 500 ms lapses, and the number of false starts), subjective feelings of sleepiness (as measured by the Stanford Sleepiness Scale; SSS), and mood (as measured by the Positive and Negative Affect Schedule; PANAS) at the five different time-points after waking. When we averaged across the two nights with each alarm condition (bedroom-based alarm vs. traditional alarm), we saw no significant difference in performance for any of the PVT metrics between the bedroom-based alarm (intervention) and traditional alarm (control) conditions. There was a significant improvement over time, with participants becoming better the longer they were awake for all PVT metrics, though this general improvement was observed for both alarm conditions. Specifically, the mean reaction time (*F*_4, 108_ = 2.58, *p* = 0.04, η_G_^2^ = 0.04), mean response speed (*F*_4, 108_ = 8.34, *p* < 0.005, η_G_^2^ = 0.06), number of false starts (*F*_4, 108_ = 3.23, *p* = 0.015, η_G_^2^ = 0.04), and percentage of 355 ms lapses (*F*_4, 108_ = 12.19, *p* < 0.005, η_G_^2^ = 0.05) and 500 ms lapses (*F*_4, 108_ = 12.18, *p* < 0.005, η_G_^2^ = 0.07) were significantly different over time with small (mean reaction time, number of false starts, percentage of 355 ms lapses) to medium (mean response time, percentage of 500 ms) effect sizes. Finally, there was no significant difference in PVT performance for any of the metrics between the intermediate and evening types.

**Figure 1:**
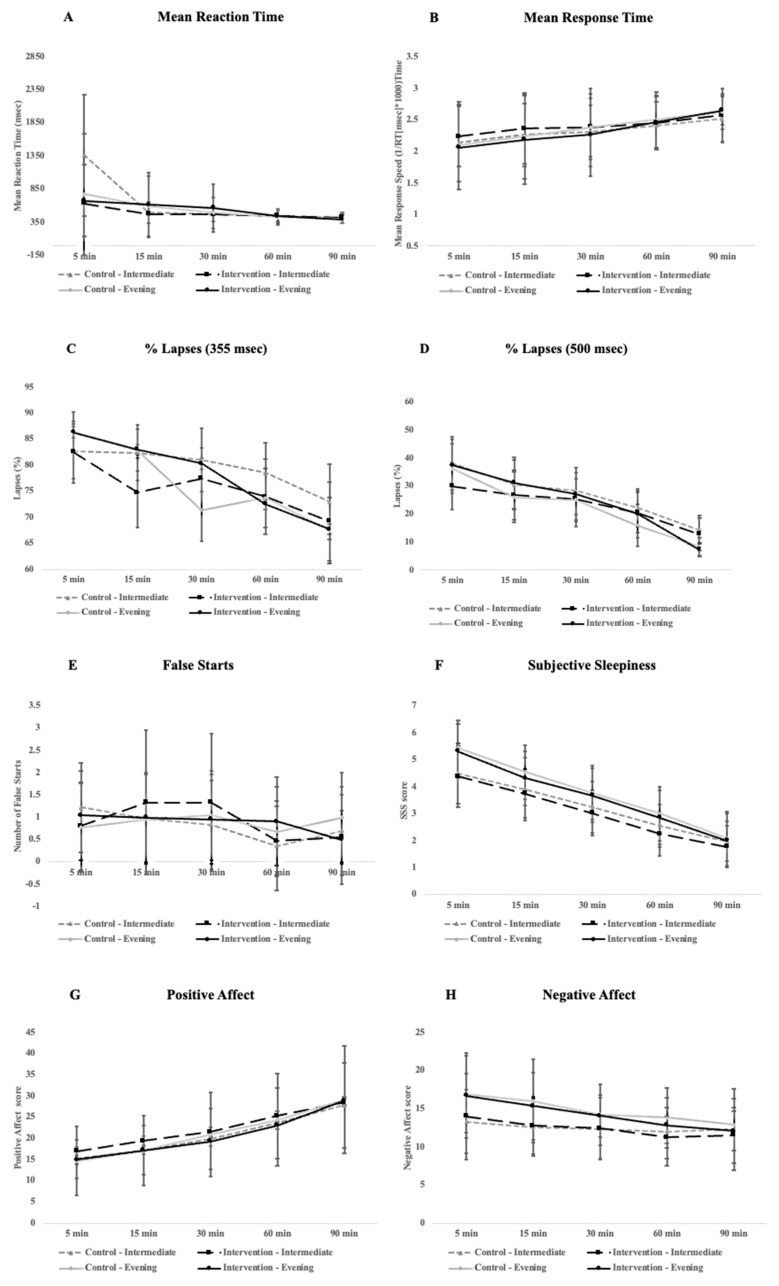
Plots for different daytime outcome measures, including PVT performance (**A**–**E**), alertness (**F**), and mood (**G**,**H**) across the 90 min cognitive testing period participants underwent immediately after waking in the morning. Error bars represent standard deviation.

For subjective feelings of sleepiness, there was a significant change over time with a large effect size *F*_4, 108_ = 138.69, *p* < 0.005, η_G_^2^ = 0.53, where perceived sleepiness dissipated the longer people were awake (see Figure 1F). However, feelings of sleepiness did not significantly differ across conditions. Moreover, the interaction between the condition and time-point was not significant. There were also no significant differences in perceived sleepiness between the intermediate and evening types.

Finally, for mood, as expected, there was a significant difference over time with a large effect size for the positive (*F*_4, 108_ = 54.73, *p* < 0.005, η_G_^2^ = 0.23) and a significant difference with a medium effect size for the negative affect (*F*_4, 108_ = 8.34, *p* < 0.005, η_G_^2^ = 0.06). However, for neither the positive nor negative affect was there a main effect for the condition or a significant interaction between the condition and time-point. Finally, there were no significant differences for either the positive or negative affect between intermediate and evening types (see Figure 1 for a full breakdown of all daytime outcomes).

### 2.2. ‘Short’ vs. ‘Long’ Alarm Experience

As the length of the alarm intervention differed between participants because of when the bedroom-based alarm was triggered for each participant, we conducted additional analyses by subdividing participants into two groups—those whose bedroom-based alarm was triggered > 15 min before their wake time (long alarm experience) and those whose bedroom-based alarm was triggered ≤ 15 min before their wake time (short alarm experience).

**Figure 2:**
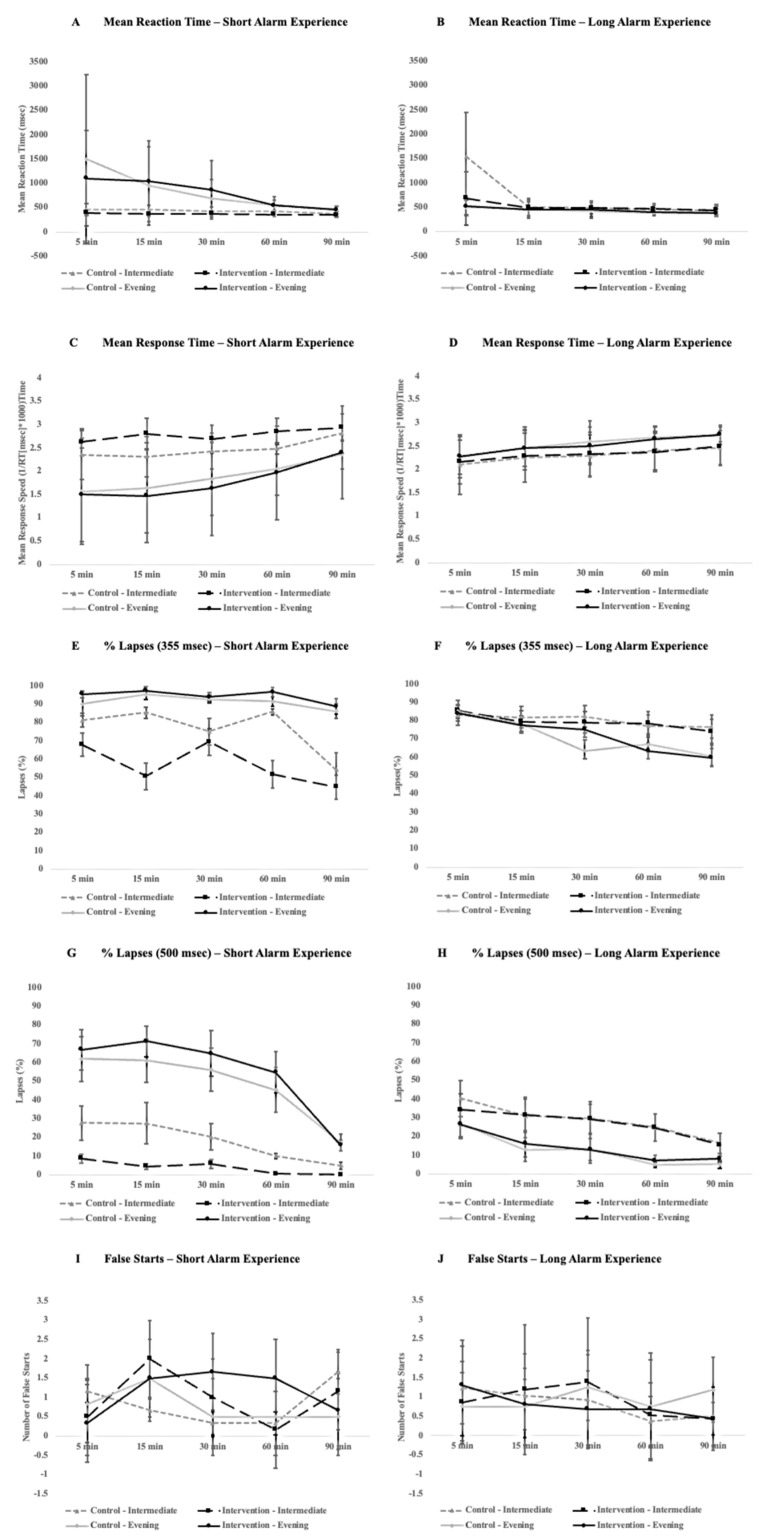
Comparison of PVT performance between short-alarm (**left**) and long-alarm (**right**) groups across a 90 min cognitive testing period that participants underwent after waking in the morning. Plots (**A**,**B**) compare the mean response time, (**C**,**D**) compare the mean response time, (**E**,**F**) compare errors of omission at a threshold of 355 ms, (**G**,**H**) compare errors of omission at a threshold of 500 ms, and (**I**,**J**) compare false alarms. Error bars represent standard deviation.

With respect to PVT performance, there were mixed impacts on performance for participants with a short-alarm experience. Specifically, for the short alarm group, there was a significant interaction with a small effect size for condition and chronotype (*F*_1, 4_ = 16.04, *p* = 0.016, η_G_^2^ = 0.04) for the mean response speed where performance improved in intermediate types after the bedroom-based intervention (*p* < 0.005, Figure 2C). There was also a significant interaction with a medium effect size for the condition and chronotype for the same group for a percentage of 355 ms lapses (*F*_1, 4_ = 9.57, *p* = 0.036, η_G_^2^ = 0.11) and significant three-way interaction with a small effect size between the time-point, chronotype, and condition (*F*_4, 16_ = 3.19, *p* = 0.042, η_G_^2^ = 0.04) with a significant decrease of 355 ms lapses for intermediate types in the bedroom-based intervention condition compared to the control condition (*p* = 0.002, Figure 2E) and increases of 355 ms lapses for evening types in the intervention condition relative to the control (*p* = 0.04, Figure 2E). In addition, evening types generally performed worse in both the intervention (*p* = 0.03) and control conditions (*p* < 0.005). Finally, for the short-alarm group there was also a significant interaction with a medium effect size between the condition and time-point for the number of false starts (*F*_4, 16_ = 3.42, *p* = 0.033, η_G_^2^ = 0.07) with the number of false starts initially dropping at the 5 min mark in the bedroom-based intervention condition relative to the control (*p =* 0.01) and then marginally increasing at the 30 min mark (*p* = 0.06, Figure 2I). All other PVT metrics were not significant for those who had a short alarm (see the plots on the left side of Figure 2 for a full breakdown of the outcome measures), with the exception of general increases in performance over time for all participants for their mean response speed (*p* = 0.016) and percentage of 500 ms lapses (*p* < 0.005).

For the participants who received a longer bedroom-based alarm experience for the intervention condition, there were some minor impacts on PVT performance. There was a significant three-way interaction with a small effect size between the condition, chronotype group, and time-point for the number of false starts (*F*_4, 84_ = 2.63, *p* = 0.04, η_G_^2^ = 0.02), with false starts marginally increasing at the 5 min time-point for evening type during the intervention condition (*p* = 0.05) and then significantly decreasing at the 90 min mark (*p* = 0.03, see Figure 2J). There was also a significant interaction between the chronotype group and testing time-point for a percentage of 355 ms lapses across both conditions (*F*_4, 84_ = 4.24, *p* = 0.004, η_G_^2^ = 0.02), with intermediate types making fewer lapses over time relative to evening types (*p* = 0.002, see Figure 2F). In general, performance as measured by the mean response speed (*p* < 0.005), percentage of 355 ms lapses (*p* < 0.005) and 500 ms lapses (*p* < 0.005), and the number of false starts (*p* = 0.002) improved over time across all participants. No other PVT metrics changed significantly for participants who had longer alarm experiences during the intervention condition (see the plots on the right side of Figure 2 for a full breakdown of outcome measures).

**Figure 3:**
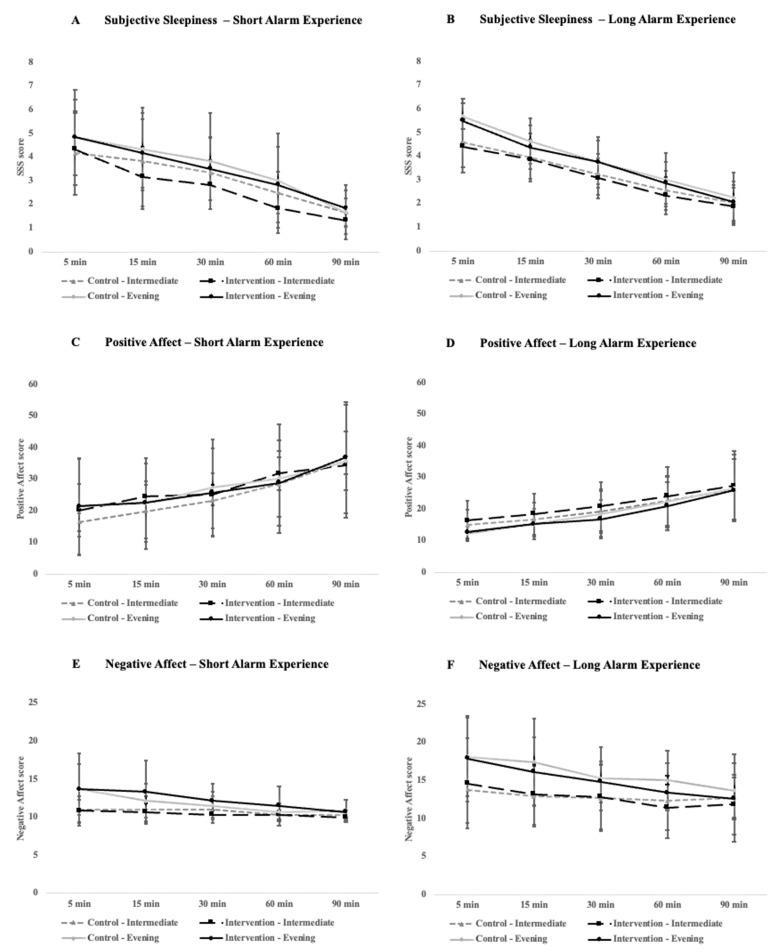
Comparison of alertness and mood ratings between the short alarm (**left**) and long alarm (**right**) groups across the 90 min cognitive testing period that participants underwent immediately after waking in the morning. Plots (**A**,**B**) compare subjective sleepiness ratings as measured by the Stanford Sleepiness Scale, (**C**,**D**) compare positive affect ratings, and (**E**,**F**) compare negative affect ratings. Positive and negative affect scores were calculated using the PANAS. Error bars represent standard deviation.

With respect to subjective perceptions of sleepiness, there were significant improvements over time across all participants in both conditions for the short-alarm (*p* < 0.005, Figure 3A) and long-alarm group (*p* < 0.005, Figure 3B). Likewise, for the positive affect, there were significant improvements over time for both the short-alarm (*p* < 0.005, Figure 3C) and long-alarm groups (*p* < 0.005, Figure 3D) that were not influenced by chronotype or condition. For the negative affect, there was a significant interaction between the condition and time-point for the participants who experienced a longer alarm time (*F*_4, 84_ = 2.51, *p* = 0.048, η_G_^2^ = 0.0048), with participants at the 60 min mark reporting marginally lower feelings of negative affect (*p* = 0.06, Figure 3F).

### 2.3. Differences in Sleep during Each Condition

To ensure that differences in PVT performance and subjective feelings were not partially influenced by potential differences in sleep quality and duration across the study period, nighttime sleep was compared between bedroom-based and traditional alarm conditions. With the exception of a marginal but not statistically significant increase in WASO (*p* = 0.065) and a statistically significant decrease in sleep efficiency (*p* = 0.042) in the bedroom-based alarm condition relative to the control condition, there were no significant differences in sleep behaviors (see Table 1 for a full breakdown of sleep measures).

## 3. Discussion

To the best of our knowledge, the current study is the first to measure the impact of a multimodal bedroom-based dawn simulation alarm—where ambient temperature, light, and sound gradually increase over a period of time prior to an individual’s scheduled wake-up time—on symptoms of sleep inertia. It has been previously demonstrated that sunrise simulation alarm clocks [31], red light exposure [30], changing sound [34,36], and manipulations to skin temperature [47] individually can have some impact on certain symptoms of sleep inertia. The current study sought to combine these various environmental elements into one intervention and measure the collective impact on sleep inertia. Moreover, as feelings of sleep inertia can be stronger when someone is woken up in a deeper stage of sleep, like SWS [4,8,9,10], we attempted to facilitate the transition to wakefulness by programming the lighting component of the multimodal bedroom-based alarm to begin when ‘light sleep’ (combined stage 1 and 2 of NREM sleep) was detected using a commercially available mattress-based sleep sensor.

Based on the promising evidence for individual environmental interventions, it was predicted that the multimodal bedroom-based alarm would decrease feelings of sleep inertia, as measured by PVT performance (mean reaction time, mean response time, percentage of 355 ms and 500 ms lapses, and a number of false starts) and subjective perceptions of sleepiness and mood, relative to a control condition where participants were woken up with a traditional alarm sound at their scheduled wake time. However, contrary to what was predicted, there was limited impact when using the multimodal bedroom-based alarm. Overall, there was no change in PVT performance (across all metrics) between the control and intervention alarm conditions. Moreover, there was no change in feelings of sleepiness, positive affect, and negative affect scores as measured by the SSS and PANAS, respectively.

While the results from the current study differed from the previous work mentioned earlier, there was one important difference. In the current study, certain aspects of the intervention, specifically the lighting component, varied across participants and were triggered when the participant entered a light stage sleep within 30 min of their scheduled wake-up time. Not only did the duration of the lighting component of the intervention differ, but also the slope of the intensity and color temperature changed. Participants who experienced a shorter lighting component during the mornings with the bedroom-based alarm also experienced a more abrupt change in lighting. To account for the differences in the duration of this intervention, we split participants into two groups: a short-alarm group (lighting duration ≤ 15 min) and a long-alarm group (lighting duration > 15 min). After participants were broken up into the following groups, there were observable differences between the two conditions, which also differed based on a person’s chronotype. Participants in the short-alarm group who were moderate evening types exhibited a higher percentage of 355 ms lapses, whereas intermediate types exhibited a better response speed and fewer 355 ms lapses. In other words, while intermediate types might have still felt the benefits of the intervention, evening types, who typically take longer to recover from feelings of sleep inertia [48], may have suffered from a more abrupt dawn simulation (lighting) experience if their alarm triggered later based on where they were in their sleep cycle. To that end, moderate evening types who experienced the longer and more gradual increase in lighting intensity and CCT during the bedroom-based alarm condition had fewer false starts at the last time-point (90 min after waking up), which further suggests that the duration and experience of the lighting may be important when designing a ‘smart’ dawn simulation intervention. Most commercially available dawn simulators are typically set to run for thirty minutes based on initial research [49], and all studies to date have used a thirty-minute dawn simulator [30,31,41,50,51,52]. The current study suggests that substantially shorter experiences might not be beneficial, and this may be one of the first studies to provide evidence of the potential efficacy of “smart alarms”, which trigger an alarm based on an individual’s sleep state. Such products are commercially available but not validated [53].

It is important to note that the intervention condition also manipulated ambient temperature and sound, and it is possible that these components may have had a marginal impact on the experience of waking up and subsequent feelings of sleep inertia. However, due to technical limitations, including our inability to manipulate temperature and continuously loop an audio track based on a person’s sleep cycle, it was not possible to properly disentangle any differential or additive effects of sound and temperature. As a result, the initiation and progressive increase in sound and temperature was the same for all participants during the bedroom-based alarm condition. Specifically, ambient temperature increased forty-five minutes before a person’s scheduled wake-up time while the audio track (nature sounds and music) began to play two minutes before the participant’s scheduled wake-up time, gradually increasing in volume over the two-minute period. By contrast, during the control condition, the portable heater was off, keeping ambient room temperature stable throughout, and participants were woken up at their scheduled wake time by a beeping alarm sound.

Therefore, given that the only differences in outcomes between the control and bedroom-based alarm conditions were observed when differences in the lighting aspect of the intervention were accounted for (i.e., short vs. long alarm groups), it is likely that the differences in outcomes that were observed were influenced primarily by the lighting component of the overall wake up experience. To that end, lighting has most consistently been shown to decrease some of the symptoms and feelings related to sleep inertia [30,31,41,50,51,52] and might potentially even protect against cardiac vulnerability after waking [54]. While some studies have shown the modest benefits of sound manipulation [34,36], the mechanisms are not clear [35]. With respect to temperature, while it is hypothesized that thermoregulation may play a role in sleep inertia [37], there are limited studies that have demonstrated a clear benefit [47]. Collectively, this could suggest that out of the list of environmental interventions that could be deployed to mitigate feelings of sleep inertia, lighting may ultimately be the most effective.

With respect to lighting as a countermeasure for sleep inertia, some research suggests that the acute alerting effects of light on performance might be dampened during the day relative to at night [45,46]. This suggests that using light as a countermeasure for sleep inertia may be more effective if used when waking at night, as is common for shift workers. Indeed, one study found that increasing blue-wavelength light was not particularly effective at countering sleep inertia after waking in the morning [55], whereas another study found that polychromatic short wavelength-enriched light exposure following nighttime awakenings did significantly improve vigilant attention and subjective measures of mood and alertness [32]. To account for the potential time-of-day differences, which may dampen the alerting benefits of part of the current study’s intervention, future studies should consider replicating the multimodal bedroom-based alarm and measure potential improvements in sleep inertia symptoms after a nighttime awakening.

With respect to sound and temperature, while we did not see differences in outcomes of sleep inertia until accounting for the differences in lighting exposure during the intervention, we cannot fully discount some minor contributions of sound or heat since we were unable to vary those elements in a meaningful way within the bedroom-based alarm condition. Future studies should continue to explore the impacts of different environmental components that may mitigate feelings of sleep inertia, including looking at physiological outcomes like changes in core body temperature or changes in sleep architecture. For example, given that ambient temperature can impact thermoregulation by influencing core body temperature [56], future studies may want to monitor core body temperature during a similar intervention to the one used in the current study. With respect to sound, future studies could measure differences in indicators of arousal using PSG data during the sound portion of the intervention in addition to correlating these arousal markers to subsequent performances on vigilance tests.

While the current study did observe changes in PVT performance after separating participants into groups based on the duration of the dawn simulation (lighting) aspect of the bedroom-based alarm, the changes were modest. Previous work has demonstrated that response speed and errors of omission (lapses), two of the measures that were impacted by the intervention in the current study, are two metrics that are particularly sensitive to deficits related to sleep [57], which adds confidence to the observations in the current study. However, mixed findings from previous work also suggest that the PVT may not necessarily be sensitive enough to fully catalog performance impairments due to sleep inertia and that other higher-order cognitive measures like working memory [3,55] should be considered. Due to tight time intervals between the initial time-points during testing the following morning, it was decided that PVT should be used given its brevity and resistance to learning effects and aptitude differences, in addition to its widespread use in studies measuring performance impairments due to sleepiness and sleep-deprived conditions [57]. However, to fully catalog any benefits of a multimodal intervention like the one in the current study, future research should measure performance across multiple cognitive tasks.

Finally, in the current study, there were some differences in sleep measures across the two conditions, with participants waking up marginally more during the night on the intervention nights and experiencing significantly worse sleep efficiency relative to control nights (86% compared to 83%). While previous work suggests that sleep loss can increase the severity of sleep inertia [11], which could ultimately wash out any benefits of this intervention, it is unclear whether the sleep efficiency score reflects objectively worse sleep or participants spending more time in bed, potentially due to being woken up earlier than expected by the intervention, specifically up to 30 min before their scheduled wake-up time. While the EarlySense Live did not allow us to calculate wake onset latency, participants were instructed to press a button indicating that they were awake as soon as they woke up and to move to the next room to start the tests, indicating that this scenario was unlikely. Nevertheless, future research could better tease out the impact of sleep by measuring sleep through polysomnography, as opposed to using a commercial sensor, which is not as accurate with limitations on sleep metrics that can be calculated.

In conclusion, the current study is the first to investigate the efficacy of a multimodal environmental bedroom-based alarm to gradually wake up participants, in contrast to using a traditional alarm, to help mitigate feelings of sleep inertia. Taking into consideration the length of the perceived intervention, specifically with respect to the lighting component, which was the only component that varied across participants, the intervention had a modest impact on feelings of sleep inertia. When taking the chronotype into account, evening types did not benefit from a shortened wake-up experience but did from the longer light-based dawn simulation component of the overall experience, suggesting that the length of the multimodal alarm, and particularly the lighting component, could influence the effectiveness of the environmental intervention. Finally, the current study might help inform the potential efficacy of “smart alarms” that activate a dawn simulation experience based on the stage of sleep someone is in.

## 4. Materials and Methods

### 4.1. Study Population

Twenty-nine adults (14 females, mean age = 27.00 ± 3.24 years) who self-reported taking longer than 30 min to wake up 60% of the working week (as determined by the Sleep Inertia Questionnaire; [58]) were recruited to participate in this study, which was approved by the local Institutional Review Board. Individuals with a history of diagnosed sleep disorders, taking medication that could impact sleep, or who were actively involved in shift work were not invited to participate. Individuals with a history of mood, psychiatric, and cardiac disorders (i.e., Raynaud’s disease, peripheral artery disease) or drug or alcohol dependency were also excluded from the study, as were women who were pregnant, intending to become pregnant, or undergoing menopause. Eligible individuals participated in a screening that included a medical chart review, disclosure of any over-the-counter (OTC) medication use and recent travel through more than three time zones, and a detailed verbal description of the study. All participants gave verbal and written informed consent for the study.

At the beginning of the study, all participants completed questionnaires (see 4.5 for detailed descriptions of questionnaires), establishing their baseline sleep quality (mean = 6.90 ± 2.68) and chronotype (18 intermediate and 11 moderate evenings). For the week prior to study participation, all participants wore actigraphy watches (Actiwatch Spectrum PRO, Philips Respironics) to track their typical sleep patterns.

Thirty-seven participants were originally recruited for the study. However, eight participants were excluded post hoc from the final analyses due to technical malfunctions during the study, which included the sleep sensor going offline during the night (three participants), the heater not turning on at the intervention onset (one participant), the building automation system resetting and canceling the scheduled intervention condition (two participants), and the app with the performance tests and surveys not working (two participants). The complete demographics for all participants are included in Table 2.

### 4.2. Overview of Study Design

The study employed a within-subjects design. Participants spent four nights in the laboratory, which was configured to look like a typical one-bedroom apartment. Two of the nights of the study were control nights, where participants were woken up by a beeping alarm sound played through a speaker (Sonos Move, Sonos Inc., Santa Barbara, CA, USA) at 50% of the maximum sound level at their designated wake time and two nights were intervention nights where participants were woken up by the multimodal bedroom intervention (see Section 4.3 for details). The order of conditions was counterbalanced across participants to control for order effects.

To simulate real-world conditions, participants were woken up at their regular weekday wake-up times, which, for everyone, was around 6:20 a.m. However, those times were kept consistent across conditions (e.g., if a participant woke up at 6 a.m. in the control condition, they were also woken up at 6 a.m. during the experimental condition). Sleep was monitored using a mattress-based sleep sensor, EarlySense Live (Early Sense Ltd., Ramat Gan, Israel).

Participants completed a vigilance test and mood and alertness ratings (see Section 4.5 for detailed descriptions of the tests and rating scales) at five different time-points each morning (5 min, 15 min, 30 min, 60 min, and 90 min) to measure changes in sleep inertia symptoms. Finally, to avoid the effects of caffeine on cognitive performance, participants were restricted from drinking caffeine until the end of the daily tests (after the fifth time-point).

### 4.3. Multimodal Bedroom-Based Alarm Intervention

**Figure 4:**
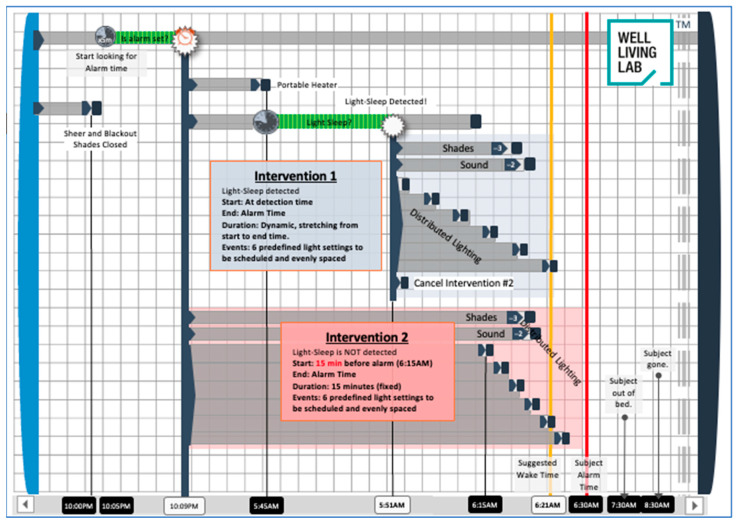
Diagram of multimodal environmental-based alarm intervention. Intervention 1 describes the scenario where light sleep was detected, and the lighting component of the intervention was initiated. Intervention 2 describes the backup scenario where light sleep was not detected, and the intervention was triggered automatically 15 min before the scheduled wake-up time.

The multimodal bedroom-based alarm intervention manipulated ambient temperature, lighting, and sound. The intervention began 45 min before the participant’s scheduled wake-up time, with a gradual increase in the ambient temperature in the room by a total of 3 °F by the end of the intervention. The rest of the intervention was triggered when the participant’s sleep sensor detected a 5 min bout of ‘light sleep’. Fifteen to thirty minutes before the participant’s scheduled wake-up time, depending on when light sleep was detected, the lighting in the bedroom gradually increased on a sigmoidal curve from 0 to 300 lux. The final correlated color temperature (CCT) in the room was approximately 4000 K. Lighting conditions were determined based on previous studies [31,41]. Three minutes before the scheduled wake-up time, the blackout shades in the room raised, allowing natural light to enter the room, and 2 min before the scheduled wake-up time, an audio recording comprised nature sounds, and music started to play through a speaker gradually increasing to 50% of the maximum sound level. In situations where light sleep was not detected within the 15 to 30 min window, the lighting portion of the intervention was triggered at the 15 min mark. In this scenario, while lighting still increased on a sigmoidal curve, the increase was more abrupt. The other sensory components of the intervention (temperature and sound) were triggered at the same time-points relative to their scheduled wake-up time. The sequence of events was scheduled to occur automatically through a building automation system (Crestron Electronics, Rockleigh, NJ, USA). See Figure 4 for a visual depiction of the intervention. Once participants had woken up, they pressed a button on a tablet (iPad, Apple Inc., Cupertino, CA, USA) by their bed to indicate they were awake, got out of bed, and went into an adjoining room to complete the PVT tests and questionnaires. Light levels in the adjoining room matched the set point at the end of the bedroom-based alarm condition. None of the participants woke up prior to the completion of the bedroom-based alarm condition or prior to the traditional alarm in the control condition.

### 4.4. Cognitive Performance, Alertness, and Mood

Participants completed performance tests and rated their perceived sleepiness and mood through a web application administered on a dedicated tablet (iPad, Apple Inc., Cupertino, CA, USA) at five different time-points (5 min, 15 min, 30 min, 60 min, and 90 min after waking up).

The first time-point was calculated from when they indicated that they were awake through the button press, which triggered a link to the testing app to be sent to the tablet in the other room.

Performance was measured using the Psychomotor Vigilance Task (PVT), a simple and brief vigilance task that has proven effective for examining circadian and homeostatic effects in sustained attention [59,60,61]. Participants were instructed to touch the screen as soon as they saw a circle appear on it, which occurred at random time intervals between 1 and 4 s. The current study used a 3 min variate of the task, which has been shown to be similarly effective for the traditional longer version of the task [62,63,64,65]. After each PVT test, participants answered questions regarding their current feelings of affect/mood from the Positive Affect Negative Affect Schedule (PANAS) [66] and alertness from the Stanford Sleepiness Scale (SSS) [67].

### 4.5. Sleep Behaviors

Before the start of the study, participants completed baseline questionnaires, which included the Morning-Eveningness Questionnaire to measure diurnal preferences [68] and the Pittsburgh Sleep Quality Index (PSQI) to assess sleep quality [69]. Daily measurements of sleep were collected using a commercially available piezo-electric sensor called the EarlySense Live (Early Sense Ltd., Ramat Gan, Israel). Heart rate, respiration rate, and movement were collected and analyzed to calculate various sleep parameters. EarlySense Live has been independently validated against polysomnography (PSG), the sleep measurement gold standard, and compared to other commercially available sleep sensors [70]. A proprietary automatic algorithm developed by EarlySense was used to classify wakefulness, sleep, and sleep stages in 30 s increments. These increments were combined across the night to calculate sleep duration, sleep onset, sleep onset latency, wake after sleep onset (WASO), and the sleep efficiency index (SEI; ratio of the total time spent asleep in the evening to the time a person spends in bed), and minutes in deep sleep (slow wave sleep), rapid eye movement (REM) sleep, and light sleep (stages 1 and 2 of non-REM sleep) [71]. Compared to polysomnography, the sensitivity for the EarlySense Live to detect sleep was 0.96, indicating a high ability to correctly detect sleep. Specificity, however, was 0.49. With respect to light sleep, sensitivity was 0.57, and specificity was 0.69, which indicates a moderate ability to correctly detect light sleep. Compared to PSG, EarlySense Live tended to underestimate light sleep and overestimate deep sleep. Comparatively speaking, all other commercially available sensors overestimate light sleep and underestimate deep sleep [70]. As a result, to avoid the likelihood of erroneously triggering the intervention during a deeper stage of sleep, we chose to use EarlySense Live for the study. Nights with irregular sleep patterns of sleep durations > 12 h were excluded from the analysis (0% of total cases).

### 4.6. Statistical Analyses

The effects of the intervention on symptoms of sleep inertia (as measured by PVT performance and SSS and PANAS scores) across time were assessed by repeated measures analysis of variance (ANOVA) with the scene (control, bedroom-based alarm intervention) and time-point (5 min, 15 min, 30 min, 60 min, and 90 min after waking) as within-subjects variables. In addition, due to chronotype-specific differences in sleep inertia recovery [48], chronotype (intermediate, moderate evening) was added as a between-subjects factor. The effect size was assessed as general eta squared (η_G_^2^). Interaction effects were further evaluated with planned comparison t-tests.

The lighting part of the alarm intervention differed based on when the initiation of the lighting sequence was triggered (with the first incidence of light sleep ~15–30 min prior to a participant’s scheduled wake time or 15 min before their scheduled wake time regardless of sleep stage), we conducted additional analyses on participants with a long alarm experience (triggered more than 15 min before their scheduled wake time) and a short alarm experience (triggered 15 min or less before their scheduled wake-up time). The performance for these two groups was measured through repeated measures analysis of variance (ANOVA) with the scene (control, bedroom-based alarm intervention) and time-point (5 min, 15 min, 30 min, 60 min, and 90 min after waking) as within-subjects variables and chronotype (intermediate, moderate evening) added as a between-subjects factor.

For PVT output measures, we assessed the mean reaction time, mean response speed (reciprocal reaction time calculated as 1/RT*1000), the number of false starts (error of commission where the participant makes a response < 100 ms), and two kinds of errors of omission (percentage of 355 ms and 500 ms lapses, [64]). For PVT metrics, we removed any outliers that were more than 2 standard deviations from the mean. For PANAS measures, we analyzed positive and negative affect as separate scores.

To account for any influence that the previous night of sleep might have had on participants’ performance the next morning, repeated-measures ANOVAs were conducted with condition (control, bedroom-based alarm intervention) as a within-subjects factor. We assessed the total sleep duration, time in bed, sleep onset, sleep onset latency, wake after sleep onset (WASO), and sleep efficiency.

All measures were averaged across the two nights of each condition, and all statistical analyses were conducted in R (Version 3.6.2). Statistical significance was defined as two-sided *p*-values < 0.05.

## Figures and Tables

**Table 1:** Differences in sleep measures.

	Control	Intervention	*p*-Value
**Sleep Duration (min)**	396.41 (47.37)	402.45 (73.67)	0.642
**Time in Bed (min)**	459.19 (67.42)	478.44 (75.44)	0.258
**Sleep Onset**	22:38 (76.55 min)	22:29 (90.54 min)	0.541
**Sleep Onset Latency (min)**	29.78 (16.43)	34.77 (22.18)	0.287
**Wake Time**	6:18 (80.89 min)	6:19 (79.59 min)	0.909
**WASO (min)**	52.42 (41.98)	64.79 (60.76)	0.065
**Sleep Efficiency (%)**	86.65 (5.93)	83.97 (10.18)	0.042 *

* indicates significance.

**Table 2:** Demographics of participants broken down using the original sample that was recruited in the first section, followed by the subsections that were used in the final analyses.

All Recruited Participants	
N = 37	Mean (SD)
**Age**	27.13 (3.97)
**Gender** **Chronotype**	19 females
**Intermediate**	24
**Moderate evening**	13
**PSQI**	6.87 (2.65)
**Usable participants after excluding for technical malfunctions**	
**N = 29**	**Mean (SD)**
**Age**	27.00 (3.24)
**Gender** **Chronotype**	14 females
**Intermediate**	18
**Moderate evening**	11
**PSQI**	6.90 (2.68)

## Data Availability

The data presented in this study are available on request from the corresponding authors. The data are not publicly available due to ethical restrictions.
